# Assessment of Noise Levels of Churches in the Kpone Katamanso Municipal Assembly in Ghana

**DOI:** 10.1155/2024/6674297

**Published:** 2024-07-18

**Authors:** Lyndon N. A. Sackey, Joel Abugre, Bernard Fei-Baffoe, Ebenezer Y. E. Amuah, Richmond Yeboah Amoako

**Affiliations:** ^1^ Department of Environmental Science Kwame Nkrumah University of Science and Technology, Kumasi, Ghana; ^2^ Envirorich Consult Ltd, P.O. Box ML 525, Mallam, Accra, Ghana

## Abstract

Different sound levels are encountered by pastors, ministers, and the whole congregation during church services, which may extend for hours, and also by people living close to these churches. This can have an adverse effect on their health. The maximum allowable noise level in worship places is 65 dB (A), according to the Ghana Standards Authority (GSA). The aim of the study was to quantify the noise pollution levels of the churches in Kpone Katamanso Municipal Assembly (KKMA) and assess the equivalent noise levels of the churches' indoor and outdoor spaces. This investigation is being conducted due to the high number of churches and the noise emanating from these churches in the Kpone Katamanso Municipality and the possible impact on human health. Thirty churches were chosen at random for the study; on Sundays between the hours of 7.30 and 12.0 pm, sound levels in churches were measured using a portable General Class 1 Meter type DSM403SD with a data logging system. MS Excel was used to evaluate the data and determine characteristics including noise exposure levels (NEL), background noise level (*L*_90_), severe noise level (*L*_10_), and equivalent noise level (*L*_eq_). The indoor equivalent noise level ranges from 74.5 dB (A) to 104.1 dB (A), and the outdoor equivalent noise level ranges from 52.6 dB (A) to 85.3 dB (A). All of the noise levels found indoors of the churches were greater than the 65 dB (A) safe allowable limit, putting the congregants and residents at risk for a variety of physiological and psychological problems.

## 1. Introduction

Noise pollution is a serious problem that has been plaguing many nations worldwide. It is especially common in markets, offices, schools, churches, mosques, and urban areas. According to Ijaiya [1], the word “noise” comes from the Latin word “nausea,” which is defined in law as “excessive, offensive, persistent, or startling sound.” Noise is defined as “unwanted sound,” which indicates sound pressure levels that are obtrusive or interfere with people's activities. Noise is defined as any undesired, unpleasant, unexpected, and louder sound above the threshold limits [2–4]. Noise may impact people's communication, health, and enjoyment of social interactions. Noise exposure has been linked to a variety of illnesses, including cardiovascular disorders, sleep difficulties, annoyance, hearing impairment, and interference with spoken communication [5].

Religious houses are springing up in the Kpone Katamanso Municipality at an alarming rate in all accessible sites, owing to the prevalent assumption that religion can provide solutions to a multitude of concerns afflicting people. Ghana allows religious freedom, yet the town's noise-related environmental impact is quite important. Being a multireligious country, Ghana's religious centers typically host congregational worship in churches as well as other settings such as fields, schools, and private houses during the day and occasionally at night. Loud public address systems contribute to high noise levels, amplifying worshippers' voices with an unbearably loud noise emanating from the religious buildings. Religious facilities that host loudspeaker competitions are another significant cause of social unrest. It is highly probable that these alterations have led to a rise in noise levels that are detrimental to the local population [6]. According to Lilly-Tariah et al. [7], churches and mosques are great places for sound to propagate, which increases the risk of noise-induced hearing loss. Many of them use loud musical instruments to enhance their musical experience, and some of them are always packed with thousands of people during their weekend services, playing loud music. The repercussions of being exposed to excessive noise levels are not well understood by a large portion of the population in this region. Even though the noise is loud and detrimental to people's health, the government has not taken much action to solve this significant public health concern. A significant contributor to hearing loss, affecting at least 5% of the global population—328 million adults and 32 million children—is exposure to excessive noise, according to the World Health Organization [8]. In addition, the WHO “United Nations Road Safety Collaboration” 2015 lists air pollution as the second most dangerous form of environmental pollution, water pollution as the most dangerous, and noise as the third most dangerous. The term “noise pollution” is now used to describe the risk of sound, the effects of which on modern development are immeasurable. Noise is a tonal component that is uncomfortable to man and more or less intolerable to him. Because of the discomfort, exhaustion, disturbances, and, in some circumstances, the pain it causes, noise has increasingly become a major environmental stressor [9]. Small rooms such as churches and other places of worship absorb a lot of sound, which raises the risk of noise-induced hearing loss. They have large speakers, bands, an organ, a chorus, and a piano, among other sound sources. It is possible to mitigate the potentially detrimental effects of sound in places of worship by incorporating “special sound controls” into the sound systems. These devices reduce the audible effect by automatically limiting the output volume or sound intensity of the systems. Hence, there is a need to assess the levels of noise emanating from these churches and put measures to reduce their activities on human health. This study aimed to assess the noise level of churches in the Kpone Katamanso Municipality.

## 2. Materials and Methods

### 2.1. Study Area

**Figure 1:**
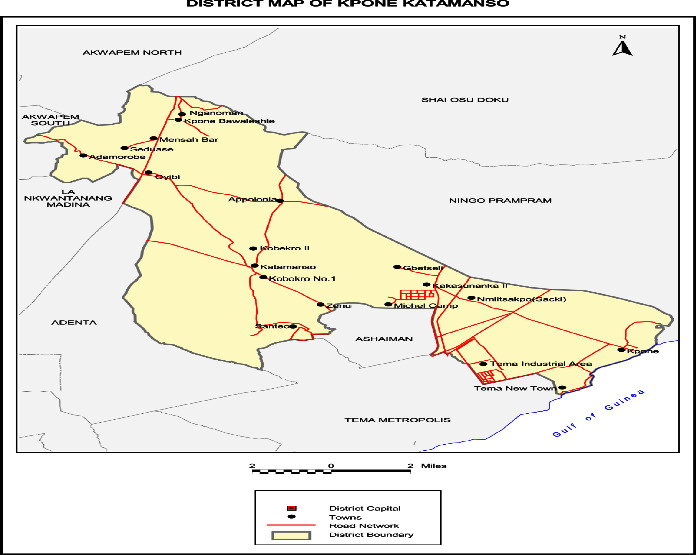
Map of the study area.

In 2012, the Legislative Instrument (L.I.) 2031 was enacted, separating the Kpone Katamanso Municipal Assembly (KKMA) from the Tema Metropolitan Assembly, situated in the eastern part of the Greater Accra Region. By the enactment of Legislative Instrument (L.I) 2271 in December 2017, the district was granted municipal status. Kpone serves as the municipal capital of the municipality, which spans from the coast to the southernmost foothills of the Akuapim Mountains. Its borders are shared by the Gulf of Guinea to the south, the Akuapim North Municipal Assembly to the north, the Adentan Municipal Assembly, the La Nkwantanang and Ashaiman Municipal Assembly, and the Tema Metropolitan Assembly to the east. The Kpone-Katamanso Municipal Assembly is only 38 kilometers from Accra, the capital city of Ghana, and falls on longitude 004′0E and latitude 50 40′ 60N (Figure 1).

### 2.2. Selection of Churches and Working Sites

The KKMA, which has a large concentration of houses of worship, is where the study was carried out. The selection criteria took into account the distribution of houses of worship, population density, and topography. For example, a purposive sample technique was used to select the churches, and the following criteria were taken into account: the church had to be Orthodox, Pentecostal, or Charismatic. The church's physical structure ought to be consistent and long-lasting rather than ephemeral.

### 2.3. Noise Level Measurement

The General Class 1 Meter type DSM403SD was used to collect the data. The noise levels of the different churches sampled were measured both indoors and outdoors in the church buildings at a distance of 20 meters from the source, to get an idea of how far the noise could travel. Before beginning the assignment, the lab calibrated the noise meter for the exercise using Standard Operating Procedures (SOPs). Using a portable General Class 1 Meter type DSM403SD with a data logging system, noise levels were recorded in situ in decibels on the A scale, or dB (A). Noise measurements are frequently “A-weighted” to account for the fact that certain sound wavelengths are thought to be exceptionally loud and insensitive to human hearing. As a result, the A scale provides the sound frequencies that human ears are most sensitive to more weight. To ascertain whether noise is within the GSA/EPA guidelines of 65 dB (A) for a place of worship, noise levels were measured for 1 hour both indoors and outdoors of the churches using the Equivalent Sound Level as a reference point. The data were logged into the equipment memory. Later, the statistical summaries were retrieved in order to be examined.

### 2.4. Sampling Size

Only the male and female age groups of 15 to 64 were considered and collected as the sample frame of the questionnaire survey, out of a total of 417,334 residents obtained from the [10]. In addition, the survey's sample size was determined using the mathematical technique developed by Yaro Yamane. A sample size of 100 of the total population was obtained. This figure was retrieved from the statistics as shown in the following equation:

(1) $$\begin{array}{c} \text{Sample size}\left(x\right)=\frac{N}{\left(1+N\right)\;E^{2}}\\ =\frac{417,334}{\left(1+417,334\right)0.1^{2}}\\ =100, \end{array}$$

where *N* is the total population and *E* is the margin of error. Population = 417334; *E* = 0.10 or 10%

Through carefully planned random selection techniques, 100 households were chosen as respondents.

### 2.5. Data Source

Data were gathered using various qualitative and quantitative techniques, such as focus group discussions (FGDs), in-depth interviews, key informant interviews, observation, and surveys. Residents' information about noise pollution at their residences was gathered using structured questionnaires. Institutional interviews were also conducted to obtain firsthand knowledge about the interventions being implemented to reduce noise pollution in Kpone Katamanso Municipal, specifically about church activities, as well as the advancements achieved and difficulties encountered by KKMA in implementing these interventions.

The following statistical summaries were computed from the recorded data and are as explained below and compared with the Ghana Standard (GS) (Table 1).

### 2.6. Data Analysis

The Statistical Package for Social Scientists (SPSS 16.0 edition) was used to code and enter the data from the questionnaire for quantitative analysis, and descriptive statistical analysis was used to analyze the data. Analyses of the field's qualitative data were conducted using in-depth justifications, narratives, quotes, and descriptions.

## 3. Results

### 3.1. Analysis of Data

There were 42 men (42%) and 58 women (58%) who participated in the study, with the latter group having a modestly larger representation (Table 2). Most of the respondents were in the age category of 20–29 years old (*n* = 37 or 37%), followed by 30–39 years (*n* = 19 or 19%), 40–49 years (*n* = 13 or 13%), 10–19, years, 50–59 years, and 60+ years recorded populations and percentages *n* = 17 or 17%, *n* = 11 or 11%, and *n* = 3 or 3%, respectively. The results show that most of the respondents 56% (*n* = 56) fall under 20 years and above. The frequency and percentage of the respondents indicated the highest education levels out of a sample of 100 people. The sample mainly comprises education levels ranging from primary to tertiary level. Most of the respondents (*n* = 66 or 66%) have obtained postsecondary education and followed by those who have obtained basic education (*n* = 25 or 25%). Respondents that had no educational qualification recorded (*n* = 9 or 9%).

The high levels of education of the population indicate that many of the respondents may be able to identify and evaluate easily what is happening in the municipal residential area. (*n* = 67 or 67%) indicated that they cannot sleep when there is much noise going on and this can result in many health implications because of the lack of sleep. Also, (*n* = 21 or 21%) of the respondents indicated that they force themselves to sleep, whereas (*n* = 11 or 11%) said they are not bothered by the noise, and only (*n* = 1 or 1%) indicated that despite high levels of noise they can still sleep soundly.

### 3.2. Indoor Noise Level Results

Indoor noise levels were obtained from 30 churches randomly sampled in the Kpone Katamanso Municipality (Table 3).

### 3.3. Outdoor Noise Level Results

Outdoor noise levels were obtained from 30 churches randomly sampled in the Kpone Katamanso Municipality (Table 4).

### 3.4. Noise Exposure Level, *L*_ex_, *T* in dB (A)

Noise exposure is important in determining the impact of noise on people's health. According to Münzel et al. [11], medical science experts believe that prolonged noise exposure can result in stress-induced conditions such as cardiovascular, diabetes, hypertension, and psychiatric issues. As a result, normal noise levels should never exceed 60 dB (A) because anything higher than it risks one's ability to hear. According to Zhang et al. [12], exposure is utilized to forecast hearing loss brought on by noise. It is obtained from the measured *L*_eq_, *t*, by making a small modification to consider the workers' hearing and the workday length. It answers the question: what would *L*_eq_, *t*, be the value of the energy that entered one's ear during *t* hours instead of during 8 hours? It is possible to compare *L*_eq_, *t*, for working days of various lengths directly by computing *L*_ex_, *T* (with a capital *T*).

The following formula, the following equation converts *L*_eq_, t, into *L*_ex_, *T*:

(2) $$\begin{array}{c} \text{Lex},T=\text{Leq},t+10\;\log\left(\frac{t}{T}\right), \end{array}$$

where *t* is the duration of the actual exposure in an hour. In the case of this work, an average of 3 h was used as the time of exposure during church service and *T* is the normalized duration, usually = 8 h.

Indoor and outdoor noise exposure levels (*L*_ex_) of churches were obtained from 30 randomly sampled churches in the Kpone Katamanso Municipality (Table 5).

### 3.5. Maximum Recommended Noise Exposure Levels

National Institute for Occupational Safety and Health (NIOSH) in 1998 established the recommended noise exposure levels, and the maximum permitted daily noise dose limit is based on a 3-dB time-intensity tradeoff, also known as the equal-energy or exchange rate rule. This means that the permitted exposure period is halved for every 3 dB increase in the noise level. For instance, people should only be exposed for four hours if the exposure level rises to 88 dB (A) from 85 dB (A).  Table 6 illustrates that the permitted exposure period doubles for each 3-dB drop in the noise level.

### 3.6. Graphical Presentation of Equivalent Noise Levels for Indoor and Outdoor

**Figure 2:**
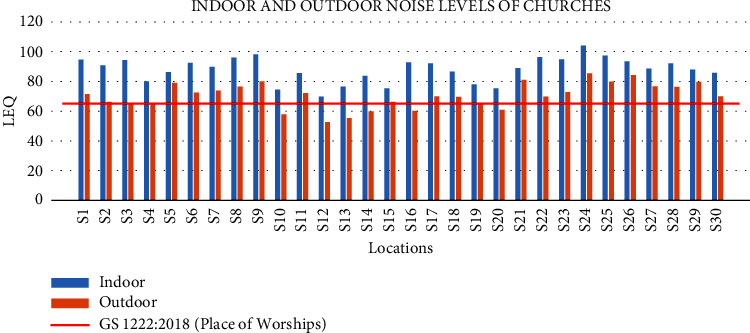
Graphical presentation of equivalent noise levels for indoors and outdoors.

From the graphical presentation of the Equivalent Noise Levels (*L*_eq_), the horizontal line (target line) that cuts through the vertical lines represents the Ghana Standard value of 65 dB (A) for places of worship (Figure 2). However, all the results from the 30 churches monitored were above the GS value for the indoor noise levels, and this means that the congregation is exposed to noise pollution with church activities. The outdoor results of the Equivalent Noise Levels (*L*_eq_) show that some of the churches were within the permissible levels of the Ghana Standard value of 65 dB (A) for places of worship, which most of these churches are orthodox churches that do not operate like the charismatic or the Pentecostal churches; therefore, they fall below the horizontal line (target line) that cuts through the vertical lines of the noise levels of the various churches monitored. However, most of the church's results were above the GS levels, and this implies noise pollution in the environments of places with churches in the municipality.

### 3.7. Indoor and Outdoor Noise Map of 30 Churches in Kpone Katamanso Municipality

**Figure 3:**
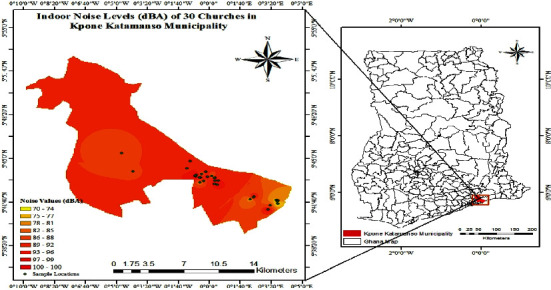
Indoor noise map of 30 churches in Kpone Katamanso Municipality.

**Figure 4:**
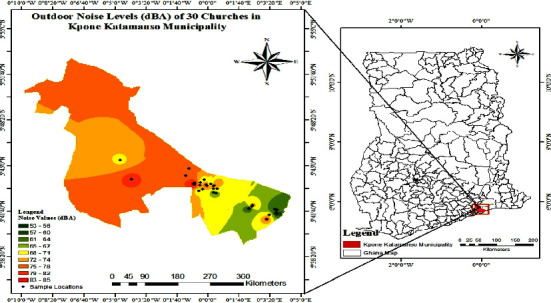
Outdoor noise map of 30 churches in Kpone Katamanso Municipality.

Indoor and outdoor noise maps of 30 churches in Kpone Katamanso Municipality are presented in Figures 3 and 4.

## 4. Discussion

### 4.1. Response to Noise Pollution

The majority (*n* = 62 or 62%) of the respondents living close to churches in the study area identified the churches as the source of noise pollution in their surroundings, mostly generated during worship and praise sessions. According to Lilly-Tariah et al. [7], churches represent an excellent sound propagation environment, posing a high risk of noise-induced loss. The results again revealed that Christianity is the dominant religion (*n* = 71 or 71%) in the municipality, which justifies why new churches are being built across the municipality rapidly. A journalist at Al Jazeera reported that Ghana's churches are part of the noise pollution problem. In the last five years, 5,000 new churches have sprung up in the capital of Ghana, Accra, which has contributed to the growing misery of residents forced to endure frequent all-night services.

Sleep is a natural process in human life that allows the body to rest and gain new strength. The majority of the respondents, 67%, indicated they find it difficult to fall asleep in a noisy environment; this exposes them to many health implications such as stress, dizziness, hearing impairment, heart attack, headache, and high blood pressure. Fry and Vyas [13] elaborated that a person exposed to noise pollution may experience difficulty falling asleep, being unable to stay asleep, and waking too early. Sounds can also reduce the depth and quality of sleep, altering the amount of rapid eye movement sleep. This can impact a person's mood and ability to concentrate. Basner et al. [5] indicated that with noise exposure, people suffer from different kinds of diseases like hearing impairment, interference with spoken communication, sleep disturbances, cardiovascular disturbances, and annoyance. Münzel et al. [11] indicated that stress and sleep disruption caused by noise pollution may result in cardiovascular disease. The World Health Organization [14] reports that prolonged exposure to environmental noise is linked to an increased risk of adverse physiological and psychological health outcomes, including cardiovascular and metabolic effects, cognitive impairment in children, and severe annoyance and sleep disturbance.

Individual judgment alone will not suffice to ensure reduced noise production. All of the public education on the negative impacts of noise pollution and the necessity of regulating noise levels will be ineffective if no steps are taken to guarantee that individuals follow the law and put the knowledge they have learned into practice. The majority (*n* = 70 or 70%) of the respondents indicated that to solve noise pollution, there should be more education about it. The Ghana Standard Authority (GS) and the Environmental Protection Agency (EPA) have established standards to protect the public from noise pollution. However, the public outcry has not received much attention from the authorities, particularly in cases involving religious institutions. This is partially due to a lack of financial, material, and human resources. This indicates that the primary issue facing the responsible authority in regulating noise levels is not the creation of new laws but rather the enforcement and application of current ones. According to Maffei and Masullo [15], awareness campaigns for people should greatly impact noise reduction.

## 5. Noise Level Generated by the Churches

### 5.1. Indoor Noise Level

The noise levels recorded for the churches indoors in the study area were high and exceeded the permissible noise of 65 dB (A) according to the Ghana Standard for a place of worship. The equivalent noise level (*L*_eq_) ranged from 69.8 dB (A) at Seventh Day Adventist–Kpone to 104.1 dB (A) at New Revival Bethel Fire Ministries (Love Cathedral Assembly) in Michel Camp with an average mean value of 88.0 dB (A) (Figure 3). Though the figures presented were all above the permissible noise level by Ghana Standard, Seventh Day Adventist at Kpone recorded the lowest equivalent noise levels (*L*_eq_) value of 69.8 dB (A), and New Revival Bethel Fire Ministries (Love Cathedral Assembly), Michel Camp, recorded the highest value of 104.1 dB (A) which implies that the impact levels are varied. The variations in the noise levels may be due to the type of instruments that are being used at the various churches during service, such as loudspeakers, bans, pianos, organs, and guitars; the Orthodox churches use fewer of these instruments, whereas the Pentecostal churches combine all during service leading to high noise emission. The noise exposure level is also a crucial factor in determining the kinds of diseases like hearing impairment, interference with spoken communication, sleep disturbances, cardiovascular disturbances, and annoyance [5].

Experts in the field of medicine have stated that prolonged exposure to noise can result in stress-related conditions such as diabetes, hypertension, and mental health issues. As a result, noise levels should never exceed 60 decibels as they pose a risk to hearing. This information is supported by Akinpelu [9]. The maximum noise level (*L*_max_) recorded for the indoor was at New Revival Bethel Fire Ministries (Love Cathedral Assembly) with a value of 118.2 dB (A), and the least noise level (*L*_min_) recorded for the indoor was at the Church of Pentecost Kpone Central with a value of 43.2 dB (A), which is below the Ghana Standards' value of 65 dB (A) for a place of worship. According to Shittu and Remi-Esan [16], the assessment of noise level in religious houses and the residents' perception of noise pollution in the Ilaro community in Nigeria indicated that all the indoor noise levels in all the religious houses were beyond the permissible limit recommended by the World Health Organization (WHO).

### 5.2. Outdoor Noise Level

The noise level monitored for all thirty (30) churches (outdoors) exceeded the permissible noise standards for a place of worship for daytime except at Seventh Day Adventist–Kpone, Haanaa SDA Church Kokompe, RCCI-Refuge Chapel International, Kpone, Touch Life Deliverance Ministry, Golf City, Golf City SDA Church, The Cathedral, and SDA Church Saki which recorded values below the permissible level of 65 dB (A) for a place of worship during the day, and most of these churches were Orthodox churches (Table 3). The equivalent noise (*L*_eq_) ranged from 52.6 dB (A) at Seventh Day Adventist–Kpone to 85.3 dB (A) at New Revival Bethel Fire Ministries (Love Cathedral Assembly) in Michel Camp with an average mean value of 70.5 dB (A) (Table 3). The maximum noise level (*L*_max_) recorded for the outdoor was at New Revival Bethel Fire Ministries (Love Cathedral Assembly) with a value of 104.1 dB (A), and the least noise level (*L*_min_) recorded for the outdoor was at SDA Church Saki with a value of 38.5 dB (A), which is below the Ghana Standards' value of 65 dB (A) for a place of worship during the day (Figure 4). Churches near roads experience increased outdoor noise due to vehicular movement, but Sundays have low vehicular movement, reducing the impact.

### 5.3. Noise Map of Indoor and Outdoor Noise Levels of Churches in Kpone Katamanso Municipality

The study analyzed spatial variation in noise pollution across 30 sites in the Kpone Katamanso Municipality. The noise level measurements were used to generate indoor and outdoor noise maps with GIS software (ArcGIS v10.8) using an Inverse Distance Weighting (IDW) interpolation method [17]. IDW estimates values at unmeasured locations based on the surrounding sampled points. This enabled mapping noise as a continuous surface across the municipality. The indoor noise map (Figure 3) displays a range of 70 dB (A) to 100 dB (A), while the outdoor map (Figure 4) shows 53 dB (A) to 85 dB (A) using color-coded decibel levels from low to high magnitudes. As per the WHO guidelines, indoor sound levels above 70 dB (A) are considered potentially hazardous [14]. Mapping facilitates spatial analysis of noise pollution patterns and hotspots of excessive exposure [18], with implications for targeting mitigation strategies to protect at-risk communities according to local variability.

This study demonstrates an effective approach combining noise sampling and interpolation mapping to elucidate the geographic distribution of this environmental health hazard. Similar methodologies may apply to global noise assessments and remediation initiatives.

Garg et al. [19] state that noise mapping is an extremely effective technique for characterizing the graphical or visual depiction of the distribution of sound levels in the study area. When constructing noise mitigation strategies for cities, this realistic assessment method for determining the environmental noise scenario in urban and metropolitan areas is helpful. A noise map often uses contours to depict the surrounding environment. The different color-coded zones that represent the noise levels' strength and frequency in the study area are depicted in these contours. In metropolitan locations, noise mapping helps in the planning of new structures.

### 5.4. Noise Exposure Level and Health Problems

There is no doubt that noise has a detrimental impact on people's health. The highest recorded noise exposure levels, indoors and outdoors, were 99.8 dB (A) and 81.8 dB (A), respectively. These levels have the potential to cause hearing damage, stress, hypertension, insomnia, distraction that lowers productivity, and a general deterioration in quality of life. Since diverse groups have different sensitivity levels to different types of noise, it is difficult to quantify the effects of religious noise. Numerous scientific studies assess the impact of religious noise on individuals. The widespread usage of loudspeakers during religious events in Kpone Katamanso presented several health problems to the urban populace. Possible side effects include vertigo, tension, sleeplessness, mental illness, raised blood pressure, hearing impairment, and nervous breakdown. 67% of the respondents responding to the impact of noise pollution indicated that they are not able to sleep when there is much noise going on, which results in many health implications because of the lack of sleep. The World Health Organization [14] states that prolonged exposure to background noise is linked to detrimental effects on one's physical and emotional well-being. The standard exposure limit for workplace noise was established in 1998 by the National Institute for workplace Safety and Health (NIOSH) (Table 6). The 8-hour time-weighted average of 85 A-weighted (dBA) decibels defines the recommended exposure limits. Exposures this high or higher are considered hazardous. This limit is based on the idea that some people will develop hearing loss due to exposure to it. Similarly, extended exposure to noise levels at or above 85 dB (A) has been linked to irreversible hearing loss, tinnitus, and difficulty understanding speech in noisy surroundings, according to Themann [20]. It is also connected to lower income, depression, heart disease, and balance problems. Workers exposed to noise and hearing impairments can be found in many different industries, although they are more prevalent in the mining, construction, and manufacturing sectors. Even though each church under investigation was built with a specific function, none featured acoustically designed rooms. This suggests that all churches are more likely to create disorienting noises, increasing the risk of sound exposure. Worshipers should watch children carefully and give extra attention since they are more susceptible to loud noises [21].

## 6. Conclusions

The purpose of the study was to measure the noise levels and examine how people who live close to churches in Kpone Katamanso Municipality perceive noise pollution. Significant noise levels were found in indoor real-time noise measurements taken from various churches in the municipality, surpassing the 65 dB (A) maximum noise level allowed by the Ghana Standards Authority for places of worship. Most of these are connected to Orthodox churches that do not employ instruments such as brass bands, which greatly add to noise during church services. Nevertheless, several of the churches' outdoor noise levels were in compliance with the GS level of 65 dB (A). Based on the data, it is clear that noise levels of 104.1 dB (A), 98.2 dB (A), 97.3 dB (A), 96.3 dB (A), and 96.0 dB (A) from the churches' indoor spaces and 86.1 dB (A), 85.3 dB (A), and 81.0 dB (A) from the churches' outdoor spaces can cause stress, heart palpitations, headaches, sleep disturbances, tension, elevated blood pressure, reduced productivity at home, and hearing impairment, all of which deteriorate one's capacity for effective communication. A study of 100 randomly distributed questionnaires assessed the impact of noise pollution on residents near churches, revealing headaches, annoyance, sleep disturbances, stress, and hearing loss as the impact of noise pollution. To improve public health and ecosystem health through effective regulation, it is important to educate the public about acceptable noise levels and to enhance the municipal task force on noise abatement.

## Figures and Tables

**Table 1:** Noise parameters measured.

Parameters	Description
*L*_EQ_	Equivalent sound level representing the average integrated sound level accumulated during the sampling period
*L*_MAX_	Maximum sound level obtained during the sampling period
*L*_MIN_	Minimum sound level obtained during the sampling period
*L*_10_	Nuisance noise level obtained during the sampling period
*L*_50_	Average noise level recorded during the sampling period
*L*_90_	Background noise level recorded during the sampling period

**Table 2:** Information on respondents.

Gender	Frequency	Percent
Male	42	42
Female	58	58
Total	100	100

Ages	Frequency	Percent

10–19	17	17
20–29	37	37
30–39	19	19
40–49	13	13
50–59	11	11
60+	3	3
Total	100	100

Education	Frequency	Percent

None	9	9
Primary	5	5
JHS/MSLC	20	20
SHS	38	38
Tertiary	28	28
Total	100	100

The impact of noise on sleep	Frequency	Percent

Cannot sleep	67	67
Sound sleep	1	1
Force to sleep	21	21
Not bothered	11	11
Total	100	100

**Table 3:** Noise levels of churches indoors, measurements were done in line with GS 1253: 2018.

S/N	Churches	Location monitored	Noise levels, dB (A)					
			*L*_eq_	*L*_max_	*L*_min_	*L*_10_	*L*_50_	*L*_90_
S1	Apostolic Church, Michel Camp, Saki	Indoor	94.6	107.5	67.9	102.4	96.4	85.1
S2	Church of Pentecost, Bethel Assembly Bediako	Indoor	90.8	114.4	57.3	100.9	91.4	79.4
S3	Church of Pentecost, Golf city	Indoor	94.2	107.4	68.6	101.2	96.9	82.0
S4	Methodist Church Ghana, Tema diocese Kpone circuit, Saki	Indoor	80.1	105.5	63.1	92.5	78.8	69.3
S5	Church of Pentecost, Great Commission Assembly, Saki	Indoor	86.2	106.2	62.6	100.8	86.2	71.0
S6	Church of Pentecost, Ebenezer Assembly, Kokompe	Indoor	92.5	112.8	60.6	106.4	95.3	74.3
S7	Global Evangelical Church, Agape Chapel Kokompe	Indoor	89.8	106.8	62.7	100.7	90.9	77.8
S8	Strong Tower Revival Ministry International, Kpone	Indoor	96.0	113.7	51.5	105.8	97.3	85.9
S9	Action Chapel International, Juda Chapel Michel Camp, Saki	Indoor	98.2	114.9	61.7	106.9	99.3	87.0
S10	Church of Christ, Kpone	Indoor	74.5	95.9	47.0	84.8	75.8	62.9
S11	Divine Apostolic Church of Ghana. Victoria Temple, Kpone	Indoor	85.6	109.0	55.6	100.1	85.3	71.1
S12	Seventh Day Adventist, Kpone	Indoor	69.8	91.8	51.1	78.7	69.3	62.0
S13	Haanaa SDA Church Kokompe	Indoor	76.4	96.3	48.5	88.0	76.4	65.6
S14	RCCI-Refuge Chapel International, Kpone	Indoor	83.7	106.2	52.4	99.7	81.7	67.1
S15	Church of Pentecost Kpone Central	Indoor	75.2	108.6	43.2	85.7	75.7	63.6
S16	Touchlife Deliverance Ministry, Golf City	Indoor	92.7	106.9	54.5	98.1	93.6	87.6
S17	Assemblies of God, Glory International Christian Center, Golf Estate	Indoor	92.2	109.4	48.4	99.8	95.0	81.6
S18	Church of God, Ghana Golf City	Indoor	86.5	105.0	56.5	95.9	85.2	74.5
S19	Golf City SDA Church, The Cathedral	Indoor	78.0	103.6	53.5	91.0	78.9	63.9
S20	SDA Church Saki	Indoor	75.2	105.6	60.7	94.2	71.5	64.9
S21	Royal House Chapel. Community 25	Indoor	88.9	108.4	56.8	96.6	89.7	79.7
S22	The Truth Chapel Ministries International. Community 25	Indoor	96.3	110.2	69.0	103.7	98.1	86.9
S23	God of Nations Church, Saki	Indoor	94.8	100.1	88.0	98.1	92.9	90.7
S24	New Revival Bethel Fire Ministries (Love Cathedral Assembly)	Indoor	104.1	118.2	73.7	110.7	108.0	91.2
S25	Church of Pentecost, Central Assembly. Michel Camp District	Indoor	97.3	112.3	79.3	105.6	97.8	88.5
S26	Solid World Chapel, Michel Camp	Indoor	93.4	97.7	86.5	95.4	93.1	92.8
S27	Apostolic Revelation Church, Kakasunanka No 1	Indoor	88.5	94.5	84.2	90.2	87.9	87.5
S28	Lighthouse Chapel Kakasunanka No 1	Indoor	92.2	94.2	91.0	92.7	92.0	91.8
S29	Divine Healers Church, Kobekro No 1	Indoor	87.9	97.0	85.1	89.1	87.6	86.4
S30	Assemblies of God Church, Kobekro 11	Indoor	85.8	99.6	83.2	86.6	85.4	84.9
Average mean value (AMV)			88.0	105.3	64.1	96.7	88.4	78.6
GS 1222: 2018 (place of worships)			65.0					

**Table 4:** Noise levels of churches outdoors, measurements were done in line with GS 1253: 2018.

S/N	Churches	Location Monitored	Noise levels, dB (A)					
			*L*_eq_	*L*_max_	*L*_min_	*L*_10_	*L*_50_	*L*_90_
S1	Apostolic Church, Michel Camp, Saki	Outdoor (20 M)	71.4	88.4	42.4	82.9	75.5	53.7
S2	Church of Pentecost, Bethel Assembly Bediako	Outdoor (20 M)	66.2	87.5	49.8	87.1	63.0	56.1
S3	Church of Pentecost, Golf city	Outdoor (20 M)	65.4	83.5	46.1	73.5	65.7	56.9
S4	Methodist Church Ghana, Tema diocese Kpone circuit, Saki	Outdoor (20 M)	65.1	83.9	46.9	77.0	66.3	51.8
S5	Church of Pentecost, Great Commission Assembly, Saki	Outdoor (20 M)	79.0	86.0	68.1	82.1	79.3	75.5
S6	Church of Pentecost, Ebenezer Assembly, Kokompe	Outdoor (20 M)	72.4	90.7	48.9	85.4	71.5	61.0
S7	Global Evangelical Church, Agape Chapel Kokompe	Outdoor (20 M)	73.7	89.6	47.4	83.1	78.6	56.9
S8	Strong Tower Revival Ministry International. Kpone	Outdoor (20 M)	76.5	93.6	48.6	85.0	77.2	67.1
S9	Action Chapel International, Juda Chapel Michel Camp, Saki	Outdoor (20 M)	80.0	93.2	56.4	86.8	80.9	72.2
S10	Church of Christ, Kpone	Outdoor (20 M)	57.8	78.7	46.3	65.3	56.9	50.9
S11	Divine Apostolic Church of Ghana. Victoria Temple, Kpone	Outdoor (20 M)	72.2	103.7	49.1	83.9	72.2	60.6
S12	Seventh Day Adventist—Kpone	Outdoor (20 M)	52.6	77.8	38.9	63.1	50.9	45.4
S13	Haanaa SDA Church Kokompe	Outdoor (20 M)	55.3	78.7	40.1	63.8	54.8	48.3
S14	RCCI-Refuge Chapel International, Kpone	Outdoor (20 M)	59.8	77.3	41.6	68.4	61.2	49.3
S15	Church of Pentecost Kpone Central	Outdoor (20 M)	66.2	89.6	42.8	76.2	67.4	54.1
S16	Touch life Deliverance Ministry, Golf City	Outdoor (20 M)	60.1	91.6	39.2	70.2	59.3	50.1
S17	Assemblies of God, Glory International Christian Center, Golf Estate	Outdoor (20 M)	70.0	93.3	42.6	82.6	70.8	54.1
S18	Church of God, Ghana Golf City	Outdoor (20 M)	69.6	96.9	43.3	86.7	68.9	52.8
S19	Golf City SDA Church, The Cathedral	Outdoor (20 M)	64.9	93.5	43.4	77.4	63.6	52.4
S20	SDA Church Saki	Outdoor (20 M)	60.9	91.9	38.5	76.7	60.5	49.0
S21	Royal House Chapel. Community 25	Outdoor (20 M)	81.0	99.5	71.3	86.6	79.2	76.2
S22	The Truth Chapel Ministries International. Community 25	Outdoor (20 M)	69.7	101.8	48.2	77.7	70.8	59.3
S23	God of Nations International Church, Saki	Outdoor (20 M)	72.7	82.5	67.4	74.0	70.1	68.2
S24	New Revival Bethel Fire Ministries (Love Cathedral Assembly)	Outdoor (20 M)	85.3	104.1	48.1	92.0	86.9	77.3
S25	Church of Pentecost, Central Assembly. Michel Camp District	Outdoor (20 M)	79.8	89.3	74.1	82.7	80.0	76.1
S26	Solid World Chapel, Michel Camp	Outdoor (20 M)	84.1	97.5	81.4	87.8	85.9	85.4
S27	Apostolic Revelation Church, Kakasunanka No 1	Outdoor (20 M)	76.6	90.2	71.0	76.3	74.9	74.5
S28	Lighthouse Chapel Kakasunanka No 1	Outdoor (20 M)	76.2	86.7	71.2	78.0	76.1	75.8
S29	Divine Healers Church, Kobekro No 1	Outdoor (20 M)	79.6	86.2	77.2	80.4	79.5	79.0
S30	Assemblies of God Church, Kobekro 11	Outdoor (20 M)	70.0	82.7	67.1	71.4	69.9	69.7
Average mean value (AMV)			70.5	89.7	53.2	78.8	70.6	62.0
GS 1222: 2018 (place of worships)			65.0					

**Table 5:** Indoor and outdoor noise exposure levels (*L*_ex_) of churches.

S/N	Churches	Noise levels, dB (A)			
		Indoor		Outdoor	
		*L*_eq_	Noise exposure levels	*L*_eq_	Noise exposure levels
S1	Apostolic Church, Michel Camp, Saki	94.6	90.3	71.4	67.1
S2	Church of Pentecost, Bethel Assembly Bediako	90.8	86.5	66.2	61.9
S3	Church of Pentecost, Golf city	94.2	89.9	65.4	61.1
S4	Methodist Church Ghana, Tema diocese Kpone circuit, Saki	80.1	75.8	65.1	60.8
S5	Church of Pentecost, Great Commission Assembly, Saki	86.2	81.9	79	74.7
S6	Church of Pentecost, Ebenezer Assembly, Kokompe	92.5	88.2	72.4	68.1
S7	Global Evangelical Church, Agape Chapel, Kokompe	89.8	85.5	73.7	69.4
S8	Strong Tower Revival Ministry International, Kpone	96.0	91.7	76.5	72.2
S9	Action Chapel International, Juda Chapel Michel Camp, Saki	98.2	93.9	80	75.7
S10	Church of Christ, Kpone	74.5	70.2	57.8	53.5
S11	Divine Apostolic Church of Ghana. Victoria Temple, Kpone	85.6	81.3	72.2	67.9
S12	Seventh Day Adventist, Kpone	69.8	65.5	52.6	48.3
S13	Haanaa SDA Church Kokompe	76.4	72.1	55.3	51
S14	RCCI-Refuge Chapel International, Kpone	83.7	79.4	59.8	55.5
S15	Church of Pentecost Kpone Central	75.2	70.9	66.2	61.9
S16	Touchlife Deliverance Ministry, Golf City	92.7	88.4	60.1	55.8
S17	Assemblies of God, Glory International Christian Center, Golf Estate	92.2	87.9	70	65.7
S18	Church of God, Ghana Golf City	86.5	82.2	69.6	65.3
S19	Golf City SDA Church, The Cathedral	78.0	73.7	64.9	60.6
S20	SDA Church Saki	75.2	70.9	60.9	56.6
S21	Royal House Chapel. Community 25	88.9	84.6	81	76.7
S22	The Truth Chapel Ministries International. Community 25	96.3	92	69.7	65.4
S23	God of Nations Church, Saki	94.8	90.5	71.7	67.4
S24	New Revival Bethel Fire Ministries (Love Cathedral Assembly)	104.1	99.8	85.3	81
S25	Church of Pentecost, Central Assembly. Michel Camp District	97.3	93	79.8	75.5
S26	Solid World Chapel, Michel Camp	93.4	89.1	86.1	81.8
S27	Apostolic Revelation Church, Kakasunanka No 1	88.5	84.2	75.6	71.3
S28	Lighthouse Chapel Kakasunanka No 1	92.2	87.9	76.2	71.9
S29	Divine Healers Church, Kobekro No 1	87.9	83.6	79.6	75.3
S30	Assemblies of God Church, Kobekro 11	85.8	81.5	70	65.7
GS 1222: 2018 (place of worships)		65.0			

**Table 6:** Maximum noise dose exposure levels recommended by the NIOSH.

Noise levels (dB)	Maximum exposure time per 24 hours
85	8 hours
88	4 hours
91	2 hours
94	1 hour
97	30 minutes
100	15 minutes
103	7.5 minutes
106	3.7 minutes
109	112 seconds
112	56 seconds
115	28 seconds
118	14 seconds
121	7 seconds
124	3 seconds
127	1 second
130–140	Less than 1 second
140	No exposure

## Data Availability

Data are available upon request.
